# State of the Patients Admitted at the Bristol Dispensary, from the 1st of November to the 1st of December, 1801

**Published:** 1802-01

**Authors:** W. D. Rolfe

**Affiliations:** Dispensary House


*State of the Patients admitted at the Bristol Dispensary, from the 1st of November to the 1st of December,* 1801.

| | | | |
|---|---|---|---|
| Anafarca | 7 | Febris Intermittens | 2 |
| Afcites | 2 | —— Puerperalis | 2 |
| Atrophia | 1 | Hæmoptœ | 1 |
| Bilious | 1 | Inflammation | 1 |
| Confumption | 3 | Ileus | 1 |
| Contufion | 1 | Peripneumonia | 7 |
| Cynanche Tonfillare | 2 | Phthifis Pulmonalis | 1 |
| Dyfentery | 6 | Pleuritis | 3 |
| Diarrhœa | 4 | Syncope | 1 |
| Dyfpepfia | 3 | Scrophula | 2 |
| Febris Simplex | 9 | Spafm | 1 |
| —— Scarlatina | 2 | Tuffis | 2 |
| —— Typhus | 32 | Vermes | 1 |

Though laft Month's return of Typhus Fevers fhould not agree with the Editor's practical experience, yet I am forry to fay, that the veracity of the ftatement mentioned is no lefs correct.

As to the other query, the Editor's knowledge might not want this piece of information, (had he only taken the trouble to read the preceding page of his own Journal,) of the paragraph I am now anfwering to, or the note of the Bath City Difpenfary.

